# Meiotic regulation of the Ndc80 complex composition and function

**DOI:** 10.1007/s00294-021-01174-3

**Published:** 2021-03-21

**Authors:** Jingxun Chen, Elçin Ünal

**Affiliations:** Department of Molecular and Cell Biology, University of California, Berkeley, CA 94720 USA

**Keywords:** Meiosis, Kinetochore, Chromosome segregation, uORF, LUTI, Transcript isoforms, Proteolysis

## Abstract

This review describes the current models for how the subunit abundance of the Ndc80 complex, a key kinetochore component, is regulated in budding yeast and metazoan meiosis. The past decades of kinetochore research have established the Ndc80 complex to be a key microtubule interactor and a central hub for regulating chromosome segregation. Recent studies further demonstrate that Ndc80 is the limiting kinetochore subunit that dictates the timing of kinetochore activation in budding yeast meiosis. Here, we discuss the molecular circuits that regulate Ndc80 protein synthesis and degradation in budding yeast meiosis and compare the findings with those from metazoans. We envision the regulatory principles discovered in budding yeast to be conserved in metazoans, thereby providing guidance into future investigations on kinetochore regulation in human health and disease.

## Introduction

The kinetochore is an evolutionarily conserved multi-subunit protein complex that mediates chromosome segregation. Since the discovery of the kinetochore in 1980s, this large protein complex has emerged as a crucial regulatory hub that directs faithful genome partitioning. Its mis-regulation now serves as a biomarker for cancer. The past 30 years of kinetochore research have illuminated the structure and function of the kinetochore. Until recently, however, relatively little is known about how the abundance of each kinetochore subunit is regulated in specialized cellular contexts, such as meiosis. In this review, we focus on the regulation of the protein abundance of one kinetochore subcomplex, the Ndc80 complex, which dictates when kinetochores are active in meiosis.

In a simplified view, kinetochore subunits are organized into two main parts: the inner and outer kinetochore. The inner kinetochore forms at the centromere, providing the foundation for the outer kinetochore to assemble. The outer kinetochore interacts with dynamic spindle microtubules. The Ndc80 complex constitutes a major subcomplex within the outer kinetochore, composed of Ndc80 (also known as Hec1 in humans), Nuf2, Spc24, and Spc25 (reviewed in Biggins 2013). The Spc24-Spc25 heterodimer links the Ndc80 complex to the inner kinetochore (Janke et al. 2001; Wigge and Kilmartin 2001; Ciferri et al. 2005; Wei et al. 2005; Hornung et al. 2011), while Ndc80-Nuf2 pair binds to microtubules through their calponin-homology domains, a protein fold commonly found in microtubule-interacting proteins (Wei et al. 2005, 2007; Ciferri et al. 2008; Alushin et al. 2010; Lampert et al. 2013). In the current model, when the inner kinetochore binds to Spc24–Spc25, the intracomplex interaction within the Ndc80 complex is inhibited, thereby promoting the binding of Ndc80-Nuf2 to microtubules (Kudalkar et al. 2015). Unique to Ndc80, its N-terminal flexible region contributes to microtubule-binding (Wei et al. 2007; Miller et al. 2008; Alushin et al. 2010, 2012; Sundin et al. 2011). This region also contains phosphorylation sites important for regulating microtubule-kinetochore attachments (Cheeseman et al. 2006; DeLuca et al. 2006, 2011; Guimaraes et al. 2008; Akiyoshi et al. 2009; Alushin et al. 2010, 2012; Umbreit et al. 2012; Zaytsev et al. 2014) and for mediating spindle-assembly checkpoint signals (McCleland et al. 2003; Kemmler et al. 2009; Aravamudhan et al. 2015; Hiruma et al. 2015; Ji et al. 2015).

Given the importance of Ndc80, it is perhaps not surprising that Ndc80 has evolved to become the linchpin subunit of the outer kinetochore in budding yeast meiosis, the specialized cell division that generates gametes. In normal meiosis, one round of DNA replication is followed by two consecutive chromosome divisions: homologous chromosomes are segregated in meiosis I, and then sister chromatids are pulled apart in meiosis II. In budding yeast, all subunits of the Ndc80 complex, with the exception of Ndc80, have constant protein levels throughout meiosis (Meyer et al. 2015; Chen et al. 2017). In meiotic prophase, Ndc80 protein levels decline, which results in the disassembly of the outer kinetochore. Shortly before the first meiotic division, Ndc80 levels increase to allow outer kinetochore re-assembly, just in time for chromosome segregation (Asakawa et al. 2005; Miller et al. 2012; Meyer et al. 2015; Chen et al. 2017, 2020). This dynamic behavior of the outer kinetochore allows two key events to take place in meiosis I. First, the kinetochores of the sister chromatid pair attach to the spindle microtubules emanating from the same spindle pole (monopolar attachment) (Meyer et al. 2018). Second, the centromeric cohesion between sister chromatids are protected during anaphase I (Miller et al. 2012). Premature expression of Ndc80 in meiotic prophase can alter chromosome segregation pattern such that sister chromatids segregate in meiosis I. This abnormal meiosis leads to both defective and reduced number of gametes (Miller et al. 2012). Therefore, a key aspect of establishing meiosis I boils down to regulating Ndc80 protein levels, which are intricately controlled by the synthesis and degradation of Ndc80 proteins in meiosis.

## Ndc80 synthesis: toggling of two functionally distinct mRNAs

To dial up or down Ndc80 abundance, meiotic yeast cells modulate Ndc80 synthesis by controlling the levels of two mRNA isoforms expressed from the *NDC80* gene (Chen et al. 2017; Chia et al. 2017). These two mRNA isoforms share the entire coding sequence of *NDC80* but vary in their 5′ end. The longer isoform, named the long undecoded transcript isoform (*NDC80*^*LUTI*^), has a 5′-extension that contains nine upstream open reading frames (uORFs). Translation of the uORFs prevents ribosomes from accessing the main ORF; consequently, *NDC80*^*LUTI*^ cannot be translated into Ndc80 protein. The shorter, canonical mRNA (*NDC80*^*ORF*^) lacks the 5′-extension and is capable of Ndc80 protein production. Rather than being a protein-coding unit, *NDC80*^*LUTI*^ serves a regulatory function such that its transcription inactivates the canonical *NDC80* promoter through co-transcriptional histone marks and nucleosome re-positioning. As a result, upregulation of *NDC80*^*LUTI*^ expression causes downregulation of Ndc80 protein synthesis and hence, downregulation of the *NDC80* gene.

**Fig. 1:**
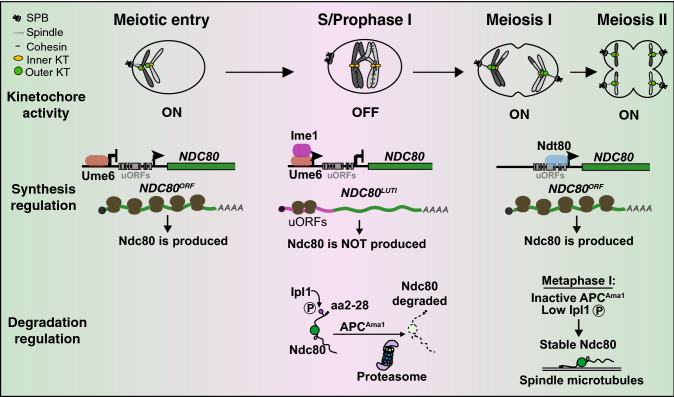
Regulation of Ndc80 abundance during budding yeast meiosis. Top: Meiotic kinetochore dynamics. The outer kinetochore becomes dissociated from the inner kinetochore during S/prophase I, thus turning off kinetochore activity. The outer kinetochore reassembles after prophase I exit and reactivates kinetochore activity for chromosome segregation during meiosis I and meiosis II. Middle: Synthesis regulation of Ndc80. During vegetative growth and premeiotic cell divisions, Ndc80 protein is synthesized from the *NDC80*^*ORF*^ transcript. Expression of the *NDC80*^*LUTI*^ transcript is inhibited by the Ume6 repressor. After meiotic entry, the master transcription factor Ime1 is expressed. Together with Ume6, the Ime1-Ume6 coactivator induces *NDC80*^*LUTI*^ expression, which turns off *NDC80*^*ORF*^ expression, leading to inhibition of Ndc80 protein synthesis. At prophase I exit, another master transcription factor Ndt80 re-expresses *NDC80*^*ORF*^, leading to re-synthesis of Ndc80 proteins. Bottom: Degradation regulation of Ndc80. During S/prophase I, the Aurora B/Ipl1 kinase phosphorylates Ndc80. Such phosphorylation, together with a short sequence (aa2-28) at Ndc80’s N-terminus, triggers Ndc80 to undergo APC^Ama1^- and proteasome-dependent degradation. After the kinetochores are properly attached to spindle microtubules in metaphase I, a time when APC^Ama1^ activity and Aurora B/Ipl1 phosphorylation are low, Ndc80 protein becomes stable to mediate chromosome segregation

Meiotic cells control the relative expression of two *NDC80* mRNA isoforms through the action of two key meiotic transcription factors. Budding yeast meiosis is induced by nutrient deprivation. Upon meiotic entry, the transcription factor Ime1 is upregulated and binds to Ume6 (Kassir et al. 1988; van Werven and Amon 2011). This Ime1-Ume6 transcription factor complex drives expression of *NDC80*^*LUTI*^ and early meiotic genes (Fig. 1). *NDC80*^*LUTI*^ transcription in turn shuts down *NDC80*^*ORF*^, which is expressed before meiosis onset, and hence inhibits Ndc80 protein synthesis. Meanwhile, Ndc80 turnover is upregulated in meiotic prophase (described in the next section). Due to the dual action of synthesis repression and degradation enhancement, the protein level of Ndc80 precipitously drops in meiotic prophase, leading to outer kinetochore disassembly. After cells exit from meiotic prophase, the mid-meiotic transcription factor Ndt80 (Xu et al. 1995; Chu and Herskowitz 1998) induces *NDC80*^*ORF*^ expression. Since *NDC80*^*ORF*^ is capable of translating Ndc80 protein, Ndc80 is rapidly resynthesized to allow assembly of the Ndc80 complex onto the inner kinetochore and reactivation of the kinetochore for mediating meiotic chromosome segregation. Therefore, the timely fluctuation of Ndc80 levels (hence outer kinetochore assembly) is ensured by coupling the expression of *NDC80*^*LUTI*^ and *NDC80*^*ORF*^ to master meiotic transcription factors.

Several mechanistic details of the *NDC80*^*LUTI*^-based repression remain unresolved. For example, nucleosomes are repositioned at the canonical *NDC80*^*ORF*^ promoter in response to *NDC80*^*LUTI*^ expression; however, the identity of the chromatin remodeler(s) responsible for this change is unknown. In addition, it is not understood how the *NDC80*^*LUTI*^-based repression mechanism is turned off after *NDC80*^*LUTI*^ expression subsides at prophase exit, the same time when the transcription factor Ndt80 re-induces *NDC80*^*ORF*^. Do the histone modifications and nucleosome positioning reset to the pre-meiotic state? If so, does the transcription factor Ndt80, or histone demethylase(s), or nucleosome remodeler(s) play a role in resetting the chromatin states? Deeper mechanistic understanding into how LUTI-based repression is established and erased will help generate models to predict LUTIs and uncover LUTI regulators in other genomes.

## Ndc80 degradation: a new function for the conserved kinase-substrate pair

In concert with the repression of Ndc80 synthesis in meiotic prophase, the premeiotic pool of Ndc80 proteins is also degraded in a regulated manner (Chen et al. 2020). Central to Ndc80 degradation is the phosphorylation of Ndc80 by Aurora B/Ipl1 (Fig. 1). Aurora B/Ipl1 is the catalytic subunit of the chromosome passenger complex, which regulates diverse cellular processes, including the assembly of bipolar spindles and kinetochore, kinetochore orientation, and cytokinesis (reviewed in Lampson and Grishchuk 2017). The functional significance of Ndc80 phosphorylation has been primarily characterized in mitotic cells: Aurora B-dependent phosphorylation weakens Ndc80’s binding to microtubules, leading to kinetochore detachment and an opportunity for the kinetochore to re-attach to microtubules in the correct orientation (reviewed in Biggins 2013; Wimbish and DeLuca 2020).

In budding yeast meiotic prophase, phosphorylated Ndc80 is a target for degradation. The Aurora B/Ipl1-dependent Ndc80 phosphorylation triggers a set of downstream events that depend on a short N-terminal segment of Ndc80 (the 2–28 residues) and the meiotic ubiquitin ligase APC^Ama1^, ultimately driving Ndc80 for proteasomal degradation (Chen et al. 2020). Notably, Ndc80 phosphorylation results in kinetochore detachment from microtubules in meiotic prophase, similar to its role in mitosis. And yet, it is unlikely that this detachment directly drives Ndc80 degradation in meiotic prophase, as microtubule depolymerization cannot rescue the defect in Ndc80 degradation resulting from Aurora B/Ipl1 depletion (Chen et al. 2020).

Besides regulating Ndc80 degradation, Aurora B/Ipl1 also plays an important role in suppressing kinetochore–microtubule interactions in meiotic prophase. Aurora B/Ipl1 does so by preventing bipolar spindle formation in meiotic prophase (Shirk et al. 2011; Kim et al. 2013). During this meiotic stage, Aurora B/Ipl1 localizes to the spindle pole bodies (SPBs, the centrosomes in yeast) and the short nuclear microtubule arrays. It has been proposed that these Aurora B/Ipl1 molecules prevent premature separation of the duplicated SPBs and formation of bipolar spindles (Shirk et al. 2011; Kim et al. 2013). By restricting microtubule activity and irreversibly abolishing the microtubule-binding site of the kinetochore through Ndc80 degradation, Aurora B/Ipl1 ensures that the kinetochore interacts with spindle microtubules only after meiotic prophase. This delayed kinetochore–microtubule interaction is required to set up a meiosis I-specific chromosome segregation pattern (Miller et al. 2012).

Several mechanistic details of Ndc80 degradation remain unresolved. For example, it is unknown how the 2–28 residues of Ndc80 mediate Ndc80 degradation. These 27 residues are necessary for Ndc80 degradation and not for Aurora B/Ipl1-dependent phosphorylation. Importantly, this region, together with the Aurora B/Ipl1 phosphorylation sites, is sufficient to induce the degradation of another kinetochore protein (Chen et al. 2020). We posit that Ndc80 phosphorylation may recruit a bipartite protein or a protein complex that binds to the 2–28 region, or Ndc80 phosphorylation may induce local conformational changes to expose the 2–28 region for factor binding. Alternatively, the 2–28 region may act in parallel with Ndc80 phosphorylation to signal for Ndc80 degradation.

Another fascinating question is which pool of Ndc80 protein is most efficiently targeted by the degradation pathway: soluble Ndc80 or the Ndc80 protein that is part of the Ndc80 complex (on or off the kinetochore)? In one model, the degradation pathway only acts on the soluble pool of Ndc80. Molecular factors would first extract Ndc80 from its complex, and Ndc80 gets degraded once it becomes soluble. In another model, Ndc80 is locally degraded at the kinetochore, while soluble Ndc80 is protected from degradation. It would be interesting to directly measure the degradation rate of soluble Ndc80 proteins versus those localized to the kinetochore to determine whether Ndc80’s kinetochore localization is required for its degradation.

Also unknown is the role of APC^Ama1^. While APC^Ama1^ is a meiosis-specific ubiquitin ligase, whether Ndc80 is a direct substrate of APC^Ama1^ remains to be tested. If Ndc80 is a direct substrate of APC^Ama1^, it would be interesting to test how Aurora B/Ipl1-dependent phosphorylation of Ndc80 affects the interaction between Ndc80 and APC^Ama1^.

Lastly, Ndc80 degradation is turned off in metaphase I through an unknown mechanism (Chen et al. 2020). We propose two non-mutually exclusive models. First, the completion of error correction may repress Ndc80 degradation. After the chromosomes correctly attach to the spindle microtubules, phosphatases remove Ndc80 phosphorylation to stabilize the microtubule attachments (reviewed in Biggins 2013). Consequently, the trigger for Ndc80 degradation (phosphorylation) would be eliminated. Second, Clb-CDK activity becomes elevated in prometaphase I, leading to inactivation of the ubiquitin ligase APC^Ama1^ (Oelschlaegel et al. 2005; Tsuchiya et al. 2011). Without APC^Ama1^, Ndc80 would be stabilized even before the completion of error correction. Future studies will be necessary to test these models.

## Regulation of the Ndc80 complex in metazoan meiosis

The basic composition and many subunits of the kinetochore are conserved between yeast and metazoans (reviewed in Musacchio and Desai 2017). The inner kinetochore of metazoans consists of the Constitutive Centromere Associated Network (CCAN). Most of the CCAN subunits have orthologs in yeast along with additional metazoan specific proteins (reviewed in Musacchio and Desai 2017). CCAN binds to centromeric chromatin throughout the cell cycle. The metazoan outer kinetochore is called the KMN network (Knl1/Mis12/Ndc80 complexes). The protein structure and core domains of the Ndc80 complex are conserved between yeast and metazoans (Wei et al. 2007). Also conserved is the role of Aurora B in correcting the erroneous attachments between the Ndc80 complex and spindle microtubules (Cheeseman et al. 2006; DeLuca et al. 2006, 2011; Alushin et al. 2012; Zaytsev et al. 2014, 2015).

One difference, however, lies in the assembly timing of the Ndc80 complex during cell division: the Ndc80 complex assembles onto CCAN only after nuclear envelope breakdown in metazoans (Gascoigne and Cheeseman 2013) while the yeast Ndc80 complex associates with the inner kinetochore during most of the mitotic cell cycle except briefly in S phase (Kitamura et al. 2007). In HeLa cells, the assembly and disassembly of the Ndc80 complex are controlled by at least two means. First, the Ndc80 complex is excluded from the nucleus until after nuclear envelope breakdown (Gascoigne and Cheeseman 2013). Second, high CDK activity promotes the Ndc80 complex to assemble on the kinetochore by phosphorylating CENP-T, which enhances the binding between CENP-T and the Ndc80 complex (Gascoigne et al. 2011; Nishino et al. 2013; Huis In't Veld et al. 2016). At mitotic exit, the declined CDK activity dampens the interaction between CENP-T and Ndc80, resulting in reduced levels of the Ndc80 complex at the kinetochore. The timely disassembly of the Ndc80 complex is required for faithful chromosome segregation in subsequent cell divisions (Gascoigne and Cheeseman 2013).

In metazoan meiosis, the Ndc80 complex also disassembles during meiotic prophase as in yeast meiosis. For example, in mice oogenesis, Nuf2 and Spc24 form puncta on chromosomes (indicating kinetochore localization) only after germinal vesicle breakdown, which corresponds to an exit from prophase I (Zhang et al. 2015, 2016). The localization pattern of Ndc80 and Spc25 is less clear since antibody staining did not show distinct puncta on chromosomes even during metaphase I and metaphase II (Sun et al. 2010, 2011). While there is no direct evidence currently, Ndc80 and Spc25 likely follow the localization pattern of Nuf2 and Spc24 because the four subunits form a complex both in vitro and in vivo (Janke et al. 2001; Wigge and Kilmartin 2001; Ciferri et al. 2005; Wei et al. 2005; Hornung et al. 2011). The mechanistic details are unknown for how mice oogenesis ensures the timely assembly of the Ndc80 complex. It would be interesting to explore how the synthesis, degradation, and nuclear localization of the Ndc80 complex are regulated during oogenesis in this organism.

In *C. elegans* oogenesis, the KMN subunits (Spc105/KNL-1 and Nuf2/HIM-10) are first detected throughout chromatin in late pachytene/diplotene and transition to chromosome surface immediately before the oocytes enter spermatheca, the time of nuclear envelope breakdown in oocytes (Howe et al. 2001; Monen et al. 2005). In metaphase I, the KMN (NDC-80, Nuf2/HIM-10, MIS-12, KNL-1, and KNL-3) cups the poleward ends of chromosomes. Surprisingly, these proteins disappear from the chromosomes after the onset of anaphase I and reappear only after metaphase II (Monen et al. 2005; Dumont et al. 2010; Davis-Roca et al. 2017). Consequently, while the outer kinetochore facilitates response to erroneous microtubule-kinetochore attachments, it is not required for segregating chromosomes in the meiosis I of *C. elegans* oocytes (Dumont et al. 2010; Muscat et al. 2015; Davis-Roca et al. 2017; Laband et al. 2017).

How the KMN disassembles during anaphase I in *C. elegans* oogenesis remains unknown. It has been shown that this disassembly requires the Y-complex nucleoporin MEL-28, which recruits protein phosphatase 1 to the kinetochore (Hattersley et al. 2016). In addition, a protein complex known as the midbivalent ring may mediate KMN disassembly. As the KMN, the midbivalent rings are also removed from chromosomes in anaphase I (Dumont et al. 2010; Muscat et al. 2015), and defective ring removal correlates with delayed kinetochore disassembly (Davis-Roca et al. 2017). Interestingly, Aurora B/AIR-2 localizes to these rings (Wignall and Villeneuve 2009; Davis-Roca et al. 2017). Inspired by the finding in yeast meiosis, we propose that Aurora B might trigger kinetochore degradation to disassemble the outer kinetochore during anaphase I in *C. elegans* oocytes.

Beyond *C. elegans* and mice oogenesis, less is known about the timing and regulation of the Ndc80 complex assembly during meiosis in other organisms. Any differences during the meiosis of different sexes (male, female, hermaphrodites, etc.) are also not well characterized. Interestingly, the localization and function of some spindle assembly checkpoint proteins display sex-specific differences in mice. For example, Mad2 localizes to kinetochores throughout meiosis I in males but is lost from kinetochores in females (Kallio et al. 2000). While oocyte meiosis requires Bub3 for accurate chromosome segregation (Li et al. 2009), spermatocyte meiosis does not (Jeganathan and van Deursen 2006). Given that the kinetochore is a key component of the spindle assembly checkpoint (reviewed in Musacchio and Desai 2017), it would be interesting to test whether the assembly and/or function of specific kinetochore subunits also differ between male and female meiosis.

## Conclusions

As a central hub for ensuring accurate chromosome segregation, the kinetochore is subjected to intricate regulation of its subunit abundance, composition, assembly, and activity. Studies of budding yeast meiosis have revealed an integrated network that regulates the synthesis and degradation of one specific kinetochore subunit, Ndc80. Since Ndc80 is a limiting component of the yeast meiotic kinetochores, its timely synthesis and degradation determines when the kinetochores are active in meiosis. Whether similar regulation of Ndc80 abundance occurs in metazoan meiosis remains to be tested. Interestingly, proteins that localize to the kinetochores (e.g., spindle assembly checkpoint proteins) seem to have sex-specific dynamics and functions. It would be interesting to examine how regulation on kinetochore subunit abundance contributes to the observed differences in male and female meiosis.

More broadly, it is of great importance to understand the regulatory mechanisms of kinetochore abundance since altered levels of kinetochore proteins have been observed in many types of cancer. For example, for many kinetochore subunits, their depletion hinders timely chromosome segregation and causes lagging chromosomes at the cleavage furrow (reviewed in Ganem and Pellman 2012; Biggins 2013). Genome instability occurs when the inner kinetochore component CENP-A is overexpressed in flies and human cells (Heun et al. 2006; Au et al. 2008; Shrestha et al. 2017). The excess CENP-A proteins, along with additional kinetochore subunits, mislocalize to non-centromeric regions. These ectopic kinetochores put chromosome arms under tension due to spindle microtubule interactions and/or reduce the protein levels of kinetochore subunits at centromeres, leading to chromosome segregation errors. Additionally, overexpression of the outer kinetochore subunits Hec1/Ndc80 or SKA1 is associated with multiple cancers and tumorigenesis (Chen et al. 1997; Chen et al. 2018; Hayama et al. 2006; Li et al. 2014; Shen et al. 2016). These studies highlight the significance of maintaining a proper level and stoichiometry of kinetochore subunits in ensuring accurate chromosome segregation. A better understanding of these regulatory pathways can provide new molecular targets for cancer treatments.

## References

[CR1] Akiyoshi B, Nelson CR, Ranish JA, Biggins S (2009). Analysis of Ipl1-mediated phosphorylation of the Ndc80 kinetochore protein in *Saccharomyces cerevisiae*. Genetics.

[CR2] Alushin GM, Ramey VH, Pasqualato S, Ball DA, Grigorieff N, Musacchio A, Nogales E (2010). The Ndc80 kinetochore complex forms oligomeric arrays along microtubules. Nature.

[CR3] Alushin GM, Musinipally V, Matson D, Tooley J, Stukenberg PT, Nogales E (2012). Multimodal microtubule binding by the Ndc80 kinetochore complex. Nat Struct Mol Biol.

[CR4] Aravamudhan P, Goldfarb AA, Joglekar AP (2015). The kinetochore encodes a mechanical switch to disrupt spindle assembly checkpoint signalling. Nat Cell Biol.

[CR5] Asakawa H, Hayashi A, Haraguchi T, Hiraoka Y (2005). Dissociation of the Nuf2-Ndc80 complex releases centromeres from the spindle-pole body during meiotic prophase in fission yeast. Mol Biol Cell.

[CR6] Au WC, Crisp MJ, DeLuca SZ, Rando OJ, Basrai MA (2008). Altered dosage and mislocalization of histone H3 and Cse4p lead to chromosome loss in *Saccharomyces cerevisiae*. Genetics.

[CR7] Biggins S (2013). The composition, functions, and regulation of the budding yeast kinetochore. Genetics.

[CR8] Cheeseman IM, Chappie JS, Wilson-Kubalek EM, Desai A (2006). The conserved KMN network constitutes the core microtubule-binding site of the kinetochore. Cell.

[CR9] Chen Y, Riley DJ, Chen PL, Lee WH (1997). HEC, a novel nuclear protein rich in leucine heptad repeats specifically involved in mitosis. Mol Cell Biol.

[CR10] Chen J, Tresenrider A, Chia M, McSwiggen DT, Spedale G, Jorgensen V, Liao H, van Werven FJ, Ünal E (2017). Kinetochore inactivation by expression of a repressive mRNA. Elife.

[CR11] Chen Y, Zhao J, Jiao Z, Wang W, Wang D, Yu X, Shi Z, Ge N, Pan Q, Xia J (2018). SKA1 overexpression is associated with poor prognosis in hepatocellular carcinoma. BMC Cancer.

[CR12] Chen J, Liao A, Powers EN, Liao H, Kohlstaedt LA, Evans R, Holly RM, Kim JK, Jovanovic M, Unal E (2020). Aurora B-dependent Ndc80 degradation regulates kinetochore composition in meiosis. Genes Dev.

[CR13] Chia M, Tresenrider A, Chen J, Spedale G, Jorgensen V, Unal E, van Werven FJ (2017). Transcription of a 5' extended mRNA isoform directs dynamic chromatin changes and interference of a downstream promoter. Elife.

[CR14] Chu S, Herskowitz I (1998). Gametogenesis in yeast is regulated by a transcriptional cascade dependent on Ndt80. Mol Cell.

[CR15] Ciferri C, De Luca J, Monzani S, Ferrari KJ, Ristic D, Wyman C, Stark H, Kilmartin J, Salmon ED, Musacchio A (2005). Architecture of the human ndc80-hec1 complex, a critical constituent of the outer kinetochore. J Biol Chem.

[CR16] Ciferri C, Pasqualato S, Screpanti E, Varetti G, Santaguida S, Dos Reis G, Maiolica A, Polka J, De Luca JG, De Wulf P (2008). Implications for kinetochore-microtubule attachment from the structure of an engineered Ndc80 complex. Cell.

[CR17] Davis-Roca AC, Muscat CC, Wignall SM (2017). Caenorhabditis elegans oocytes detect meiotic errors in the absence of canonical end-on kinetochore attachments. J Cell Biol.

[CR18] DeLuca JG, Gall WE, Ciferri C, Cimini D, Musacchio A, Salmon ED (2006). Kinetochore microtubule dynamics and attachment stability are regulated by Hec1. Cell.

[CR19] DeLuca KF, Lens SM, DeLuca JG (2011). Temporal changes in Hec1 phosphorylation control kinetochore-microtubule attachment stability during mitosis. J Cell Sci.

[CR20] Dumont J, Oegema K, Desai A (2010). A kinetochore-independent mechanism drives anaphase chromosome separation during acentrosomal meiosis. Nat Cell Biol.

[CR21] Ganem NJ, Pellman D (2012). Linking abnormal mitosis to the acquisition of DNA damage. J Cell Biol.

[CR22] Gascoigne KE, Cheeseman IM (2013). CDK-dependent phosphorylation and nuclear exclusion coordinately control kinetochore assembly state. J Cell Biol.

[CR23] Gascoigne KE, Takeuchi K, Suzuki A, Hori T, Fukagawa T, Cheeseman IM (2011). Induced ectopic kinetochore assembly bypasses the requirement for CENP-A nucleosomes. Cell.

[CR24] Guimaraes GJ, Dong Y, McEwen BF, Deluca JG (2008). Kinetochore-microtubule attachment relies on the disordered N-terminal tail domain of Hec1. Curr Biol.

[CR25] Hattersley N, Cheerambathur D, Moyle M, Stefanutti M, Richardson A, Lee KY, Dumont J, Oegema K, Desai A (2016). A nucleoporin docks protein phosphatase 1 to direct meiotic chromosome segregation and nuclear assembly. Dev Cell.

[CR26] Hayama S, Daigo Y, Kato T, Ishikawa N, Yamabuki T, Miyamoto M, Ito T, Tsuchiya E, Kondo S, Nakamura Y (2006). Activation of CDCA1-KNTC2, members of centromere protein complex, involved in pulmonary carcinogenesis. Cancer Res.

[CR27] Heun P, Erhardt S, Blower MD, Weiss S, Skora AD, Karpen GH (2006). Mislocalization of the Drosophila centromere-specific histone CID promotes formation of functional ectopic kinetochores. Dev Cell.

[CR28] Hiruma Y, Sacristan C, Pachis ST, Adamopoulos A, Kuijt T, Ubbink M, von Castelmur E, Perrakis A, Kops GJ (2015). Competition between MPS1 and microtubules at kinetochores regulates spindle checkpoint signaling. Science.

[CR29] Hornung P, Maier M, Alushin GM, Lander GC, Nogales E, Westermann S (2011). Molecular architecture and connectivity of the budding yeast Mtw1 kinetochore complex. J Mol Biol.

[CR30] Howe M, McDonald KL, Albertson DG, Meyer BJ (2001). HIM-10 is required for kinetochore structure and function on *Caenorhabditis elegans* holocentric chromosomes. J Cell Biol.

[CR31] Huis In't Veld PJ, Jeganathan S, Petrovic A, Singh P, John J, Krenn V, Weissmann F, Bange T, Musacchio A (2016). Molecular basis of outer kinetochore assembly on CENP-T. Elife.

[CR32] Janke C, Ortiz J, Lechner J, Shevchenko A, Shevchenko A, Magiera MM, Schramm C, Schiebel E (2001). The budding yeast proteins Spc24p and Spc25p interact with Ndc80p and Nuf2p at the kinetochore and are important for kinetochore clustering and checkpoint control. EMBO J.

[CR33] Jeganathan KB, van Deursen JM (2006). Differential mitotic checkpoint protein requirements in somatic and germ cells. Biochem Soc Trans.

[CR34] Ji Z, Gao H, Yu H (2015). Kinetochore attachment sensed by competitive Mps1 and microtubule binding to Ndc80C. Science.

[CR35] Kallio M, Eriksson JE, Gorbsky GJ (2000). Differences in spindle association of the mitotic checkpoint protein Mad2 in mammalian spermatogenesis and oogenesis. Dev Biol.

[CR36] Kassir Y, Granot D, Simchen G (1988). IME1, a positive regulator gene of meiosis in S. cerevisiae. Cell.

[CR37] Kemmler S, Stach M, Knapp M, Ortiz J, Pfannstiel J, Ruppert T, Lechner J (2009). Mimicking Ndc80 phosphorylation triggers spindle assembly checkpoint signalling. EMBO J.

[CR38] Kim S, Meyer R, Chuong H, Dawson DS (2013). Dual mechanisms prevent premature chromosome segregation during meiosis. Genes Dev.

[CR39] Kitamura E, Tanaka K, Kitamura Y, Tanaka TU (2007). Kinetochore microtubule interaction during S phase in *Saccharomyces cerevisiae*. Genes Dev.

[CR40] Kudalkar EM, Scarborough EA, Umbreit NT, Zelter A, Gestaut DR, Riffle M, Johnson RS, MacCoss MJ, Asbury CL, Davis TN (2015). Regulation of outer kinetochore Ndc80 complex-based microtubule attachments by the central kinetochore Mis12/MIND complex. Proc Natl Acad Sci USA.

[CR41] Laband K, Le Borgne R, Edwards F, Stefanutti M, Canman JC, Verbavatz JM, Dumont J (2017). Chromosome segregation occurs by microtubule pushing in oocytes. Nat Commun.

[CR42] Lampert F, Mieck C, Alushin GM, Nogales E, Westermann S (2013). Molecular requirements for the formation of a kinetochore-microtubule interface by Dam1 and Ndc80 complexes. J Cell Biol.

[CR43] Lampson MA, Grishchuk EL (2017). Mechanisms to avoid and correct erroneous kinetochore-microtubule attachments. Biology (Basel).

[CR44] Li M, Li S, Yuan J, Wang ZB, Sun SC, Schatten H, Sun QY (2009). Bub3 is a spindle assembly checkpoint protein regulating chromosome segregation during mouse oocyte meiosis. PLoS ONE.

[CR45] Li J, Xuan JW, Khatamianfar V, Valiyeva F, Moussa M, Sadek A, Yang BB, Dong BJ, Huang YR, Gao WQ (2014). SKA1 over-expression promotes centriole over-duplication, centrosome amplification and prostate tumourigenesis. J Pathol.

[CR46] McCleland ML, Gardner RD, Kallio MJ, Daum JR, Gorbsky GJ, Burke DJ, Stukenberg PT (2003). The highly conserved Ndc80 complex is required for kinetochore assembly, chromosome congression, and spindle checkpoint activity. Genes Dev.

[CR47] Meyer RE, Chuong HH, Hild M, Hansen CL, Kinter M, Dawson DS (2015). Ipl1/Aurora-B is necessary for kinetochore restructuring in meiosis I in *Saccharomyces cerevisiae*. Mol Biol Cell.

[CR48] Meyer RE, Brown J, Beck L, Dawson DS (2018). Mps1 promotes chromosome meiotic chromosome biorientation through Dam1. Mol Biol Cell.

[CR49] Miller SA, Johnson ML, Stukenberg PT (2008). Kinetochore attachments require an interaction between unstructured tails on microtubules and Ndc80(Hec1). Curr Biol.

[CR50] Miller MP, Unal E, Brar GA, Amon A (2012). Meiosis I chromosome segregation is established through regulation of microtubule-kinetochore interactions. Elife.

[CR51] Monen J, Maddox PS, Hyndman F, Oegema K, Desai A (2005). Differential role of CENP-A in the segregation of holocentric *C. elegans* chromosomes during meiosis and mitosis. Nat Cell Biol.

[CR52] Musacchio A, Desai A (2017). A molecular view of kinetochore assembly and function. Biology (Basel).

[CR53] Muscat CC, Torre-Santiago KM, Tran MV, Powers JA, Wignall SM (2015). Kinetochore-independent chromosome segregation driven by lateral microtubule bundles. Elife.

[CR54] Nishino T, Rago F, Hori T, Tomii K, Cheeseman IM, Fukagawa T (2013). CENP-T provides a structural platform for outer kinetochore assembly. EMBO J.

[CR55] Oelschlaegel T, Schwickart M, Matos J, Bogdanova A, Camasses A, Havlis J, Shevchenko A, Zachariae W (2005). The yeast APC/C subunit Mnd2 prevents premature sister chromatid separation triggered by the meiosis-specific APC/C-Ama1. Cell.

[CR56] Shen L, Yang M, Lin Q, Zhang Z, Miao C, Zhu B (2016). SKA1 regulates the metastasis and cisplatin resistance of non-small cell lung cancer. Oncol Rep.

[CR57] Shirk K, Jin H, Giddings TH, Winey M, Yu HG (2011). The Aurora kinase Ipl1 is necessary for spindle pole body cohesion during budding yeast meiosis. J Cell Sci.

[CR58] Shrestha RL, Ahn GS, Staples MI, Sathyan KM, Karpova TS, Foltz DR, Basrai MA (2017). Mislocalization of centromeric histone H3 variant CENP-A contributes to chromosomal instability (CIN) in human cells. Oncotarget.

[CR59] Sun SC, Lee SE, Xu YN, Kim NH (2010). Perturbation of Spc25 expression affects meiotic spindle organization, chromosome alignment and spindle assembly checkpoint in mouse oocytes. Cell Cycle.

[CR60] Sun SC, Zhang DX, Lee SE, Xu YN, Kim NH (2011). Ndc80 regulates meiotic spindle organization, chromosome alignment, and cell cycle progression in mouse oocytes. Microsc Microanal.

[CR61] Sundin LJ, Guimaraes GJ, Deluca JG (2011). The NDC80 complex proteins Nuf2 and Hec1 make distinct contributions to kinetochore-microtubule attachment in mitosis. Mol Biol Cell.

[CR62] Tsuchiya D, Gonzalez C, Lacefield S (2011). The spindle checkpoint protein Mad2 regulates APC/C activity during prometaphase and metaphase of meiosis I in *Saccharomyces cerevisiae*. Mol Biol Cell.

[CR63] Umbreit NT, Gestaut DR, Tien JF, Vollmar BS, Gonen T, Asbury CL, Davis TN (2012). The Ndc80 kinetochore complex directly modulates microtubule dynamics. Proc Natl Acad Sci USA.

[CR64] van Werven FJ, Amon A (2011). Regulation of entry into gametogenesis. Philos Trans R Soc Lond B Biol Sci.

[CR65] Wei RR, Sorger PK, Harrison SC (2005). Molecular organization of the Ndc80 complex, an essential kinetochore component. Proc Natl Acad Sci USA.

[CR66] Wei RR, Al-Bassam J, Harrison SC (2007). The Ndc80/HEC1 complex is a contact point for kinetochore-microtubule attachment. Nat Struct Mol Biol.

[CR67] Wigge PA, Kilmartin JV (2001). The Ndc80p complex from *Saccharomyces cerevisiae* contains conserved centromere components and has a function in chromosome segregation. J Cell Biol.

[CR68] Wignall SM, Villeneuve AM (2009). Lateral microtubule bundles promote chromosome alignment during acentrosomal oocyte meiosis. Nat Cell Biol.

[CR69] Wimbish RT, DeLuca JG (2020). Hec1/Ndc80 tail domain function at the kinetochore-microtubule interface. Front Cell Dev Biol.

[CR70] Xu L, Ajimura M, Padmore R, Klein C, Kleckner N (1995). NDT80, a meiosis-specific gene required for exit from pachytene in *Saccharomyces cerevisiae*. Mol Cell Biol.

[CR71] Zaytsev AV, Sundin LJ, DeLuca KF, Grishchuk EL, DeLuca JG (2014). Accurate phosphoregulation of kinetochore-microtubule affinity requires unconstrained molecular interactions. J Cell Biol.

[CR72] Zaytsev AV, Mick JE, Maslennikov E, Nikashin B, DeLuca JG, Grishchuk EL (2015). Multisite phosphorylation of the NDC80 complex gradually tunes its microtubule-binding affinity. Mol Biol Cell.

[CR73] Zhang T, Zhou Y, Qi ST, Wang ZB, Qian WP, Ouyang YC, Shen W, Schatten H, Sun QY (2015). Nuf2 is required for chromosome segregation during mouse oocyte meiotic maturation. Cell Cycle.

[CR74] Zhang T, Zhou Y, Wang HH, Meng TG, Guo L, Ma XS, Shen W, Schatten H, Sun QY (2016). Spc24 is required for meiotic kinetochore-microtubule attachment and production of euploid eggs. Oncotarget.

